# Sensorimotor Modulation Differs with Load Type during Constant Finger Force or Position

**DOI:** 10.1371/journal.pone.0108058

**Published:** 2014-09-18

**Authors:** Hikari Kirimoto, Hiroyuki Tamaki, Makoto Suzuki, Takuya Matsumoto, Kazuhiro Sugawara, Syo Kojima, Hideaki Onishi

**Affiliations:** 1 Institute for Human Movement and Medical Sciences, Niigata University of Health and Welfare, Niigata, Japan; 2 School of Allied Health Sciences, Kitasato University, Kanagawa, Japan; 3 Graduate School of Health and Welfare, Niigata University of Health and Welfare, Niigata, Japan; 4 Tokyo Bay Rehabilitation Hospital, Chiba, Japan; Duke University, United States of America

## Abstract

During submaximal isometric contraction, there are two different load types: production of a constant force against a rigid restraint (force task), and maintenance of position against a constant load (position task). Previous studies reported that the time to task failure during a fatigue task was twice as long in the force task compared with the position task. Sensory feedback processing may contribute to these differences. The purpose of the current study was to determine the influence of load types during static muscle contraction tasks on the gating effect, i.e., attenuation of somatosensory-evoked potentials (SEPs) and the cortical silent period (cSP). Ten healthy subjects contracted their right first dorsal interosseus muscle by abducting their index finger for 90 s, to produce a constant force against a rigid restraint that was 20% of the maximum voluntary contraction (force task), or to maintain a constant position with 10° abduction of the metacarpophalangeal joint against the same load (position task). Somatosensory evoked potentials (SEPs) were recorded from C3′ by stimulating either the right ulnar or median nerve at the wrist while maintaining contraction. The cortical silent period (cSP) was also elicited by transcranial magnetic stimulation. Reduction of the amplitude of the P45 component of SEPs was significantly larger during the position task than during the force task and under control rest conditions when the ulnar nerve, but not the median nerve, was stimulated. The position task had a significantly shorter cSP duration than the force task. These results suggest the need for more proprioceptive information during the position task than the force task. The shorter duration of the cSP during the position task may be attributable to larger amplitude of heteronymous short latency reflexes. Sensorimotor modulations may differ with load type during constant finger force or position tasks.

## Introduction

Muscle activities involving production of a constant force by pulling against a noncompliant restraint (force task) or maintenance of a constant limb angle while supporting an equivalent inertial load (position task), seem to involve different neural control mechanisms despite both tasks generating a similar net muscle torque [1], [2]. Many previous studies have reported that the time to task failure during a fatigue task is twice as long in the force task compared with the position task. A number of researchers have demonstrated that this unique phenomenon could occur with elbow flexion [3]–[10], index finger abduction [11], knee extension [12], dorsiflexion [13] and wrist extension [14]. Some studies have reported that position tasks produce an increased amplitude of short latency reflexes (SLR) compared with force tasks [1], [15], [16]. In addition, it has been reported that heteronymous monosynaptic Ia facilitation was greater and homonymous inhibition was depressed during position tasks [14], [17], [18], and it was thought that the greater reflex amplitude during position tasks was attributable to lower levels of presynaptic inhibition of Ia afferents [14], [19]. Furthermore, the rate at which motor units are recruited is greater during position tasks than force tasks [3], [9], [20]. These lower levels of presynaptic inhibition of Ia afferents and more rapid recruitment of the motor unit pool may contribute to the shorter time to failure when a compliant load is supported in a position task [19].

Recent studies on the neurophysiological mechanisms of the difference in time to task failure between force and position tasks have provided little information on the role of the central nervous system. Sensorimotor modulation is the process by which the motor system continuously elaborates sensory afferents in order to enhance the execution of fine motor activities [21]. Many studies have examined this using attenuation of somatosensory evoked potentials (SEPs) during voluntary movement, which is known as ‘gating’ [22], [23]. The physiologic importance of gating is to prevent irrelevant afferent inputs during movement from reaching consciousness [23]–[25], and this regulation of sensitivity to external sensory stimuli may be important in the execution of precise movements [26]–[29]. Previous studies also demonstrated that muscle stretch receptors and Ia afferents play an important role in regulating the SEP gating induced by movement [24], [25], [28], [29].

Since the first demonstration of transcranial magnetic stimulation (TMS) [30], the technique has been widely adopted to transiently alter neural excitatory/inhibitory inputs to the corticospinal tract. It is known that voluntary muscle contraction of small hand muscles greatly enhances the motor evoked potentials (MEPs) induced by transcranial magnetic stimulation (TMS). The amplitudes of MEPs vary with the intensity of different voluntary muscle contractions, which is associated with different excitability changes in the primary motor cortex (M1) [31]–[33]. Since a similar net muscle torque is exerted during both position and force tasks, the amplitude of MEPs does not differ during the two tasks [10], [33]. This indicates that when comparable α motor neurons are recruited during the two tasks, the voluntary drive seems to produce similar activation of pyramidal neurons in M1 or motor neuron pools, irrespective of the load type. Our recent study [34] and other previous studies [35]–[37] found that the duration of the cortical silent period (cSP) following TMS can be used to indicate the level of inhibition in the motor cortex during contraction. Moreover, MEPs and cSP are generated by different mechanisms [34], [38], [39]. Binder et al. [40] reported that in vibrating muscles (i.e., enhanced Ia afferent activity), the cSP duration tended to be shorter, whereas the amplitude of MEPs remained unchanged. Thus, we hypothesized that a difference in the amplitude of heteronymous SLRs between position and force tasks may affect attenuation of SEPs and the duration of the cSP. However, whether or not attenuation of SEP amplitude and the duration of the cSP differ with load type during submaximal isometric contraction has not been fully elucidated.

Therefore, the aim of this study was to determine whether sensorimotor modulation differs with the load type during constant finger force or position tasks, and this was accomplished by investigating the gating of SEPs, the amplitude of MEPs and the duration of the cSP in response to TMS.

## Materials and Methods

### Subjects

Participants in this study comprised 10 healthy individuals (9 males, 1 female; age range, 20–38 years), none of whom were receiving medical treatment for any condition. Based on administration of the Oldfield [41] inventory, the handedness scores of all subjects ranged from 0.9 to 1.0 (strongly right-handed). All participants provided written, informed consent to participate in the study, which was approved by the ethics committee of the Niigata University of Health and Welfare. All experimental procedures were approved by the same committee. This study was performed in accordance with the Declaration of Helsinki.

### Experimental Apparatus

**Figure 1:**
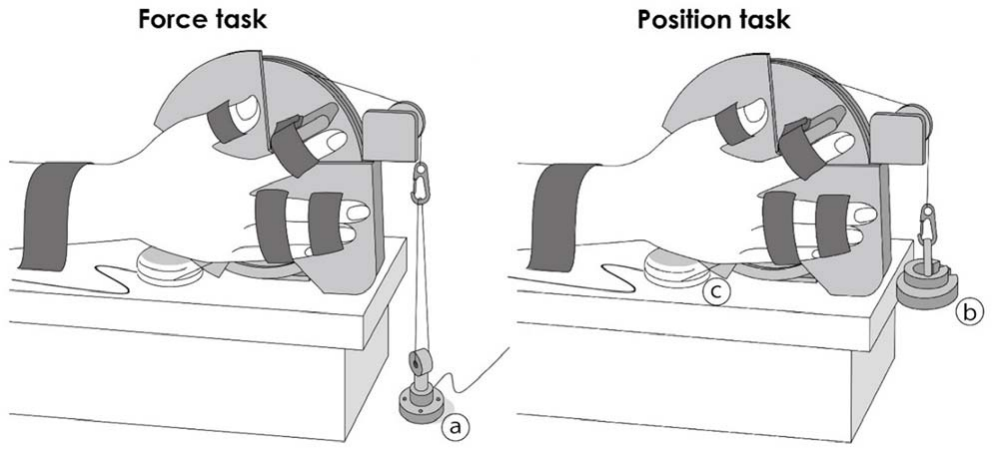
Illustration of the experimental setup for the force task (A) and the position task (B). Each subject was seated upright with the right hand positioned in the custom apparatus, and faced a monitor for visual feedback. The custom-designed device consisted of a wheel connected to a force transducer (a) or inertial load (b) by means of a pulley and nylon line. The index finger was attached to a bar that was connected to the wheel so that the rotational axis of the metacarpophalangeal joint approximated that of the wheel. Subjects were required to match either a target force equal to 20% of their maximal force by pushing up against a rigid bar (force task (A)), or a target position corresponding to 10° abduction of the metacarpophalangeal joint while supporting an equivalent load suspended from the index finger (position task (B)). The abduction angle of the metacarpophalangeal joint during the position task was measured with a wire-type displacement meter (c) attached to the wheel.

Subjects were seated upright with the right hand positioned in the custom-designed apparatus (Fig. 1) when performing force and position tasks with the index finger. The custom-designed device consisted of a wheel connected to a force transducer (TT-FR, TEAC, Tokyo, Japan) or isoinertial load by means of a pulley and nylon line. The index finger was attached to a bar that was connected to the wheel so that the rotational axis of the metacarpophalangeal joint approximated that of the wheel [42]. In addition, the index finger was restrained with a bar to maintain the interphalangeal joints in full extension, and to allow only abduction–adduction with respect to the metacarpophalangeal joint. Subjects were required to match either a target force equal to 20% of their maximal force by pushing up against a rigid bar (force task), or a target position corresponding to 10° abduction of the metacarpophalangeal joint while supporting an equivalent load suspended from the index finger (position task). The abduction angle of the metacarpophalangeal joint during the position task was measured with a wire-type displacement meter (MTA-5E-5KW-MB, Celesco Transducer Products Inc, Chatsworth, California, USA) attached to the wheel. Visual feedback was provided on a monitor during both tasks, at a gain equal to 2.5%/cm of the maximal performance range, operationally defined as a maximal voluntary contraction (MVC) for the force task and full range of motion about the metacarpophalangeal joint for the position task [11]. The right upper arm was slightly abducted (10–20°), the elbow joint was flexed to 110° and the forearm positioned midway between pronation and supination. The thumb was restrained at 45° abduction and the third, fourth and fifth digits were secured with the metacarpophalangeal and interphalangeal joints fully extended. The position of the index finger and restraints for the other fingers and wrist were individually adjusted and marked for each subject, using rulers that were permanently attached to the experimental apparatus, to minimize the variation in positioning of the arm and hand across sessions.

Signals from the force transducer and wire-type displacement meter were low-pass filtered (50 Hz) and digitized at 10 KHz (PowerLab/8sp 16 bit, AD Instruments, Bella Vista, Australia). The data were recorded and stored for off-line analysis (LabChart 7.3, AD Instruments) on a personal computer.

### Protocol

**Figure 2:**
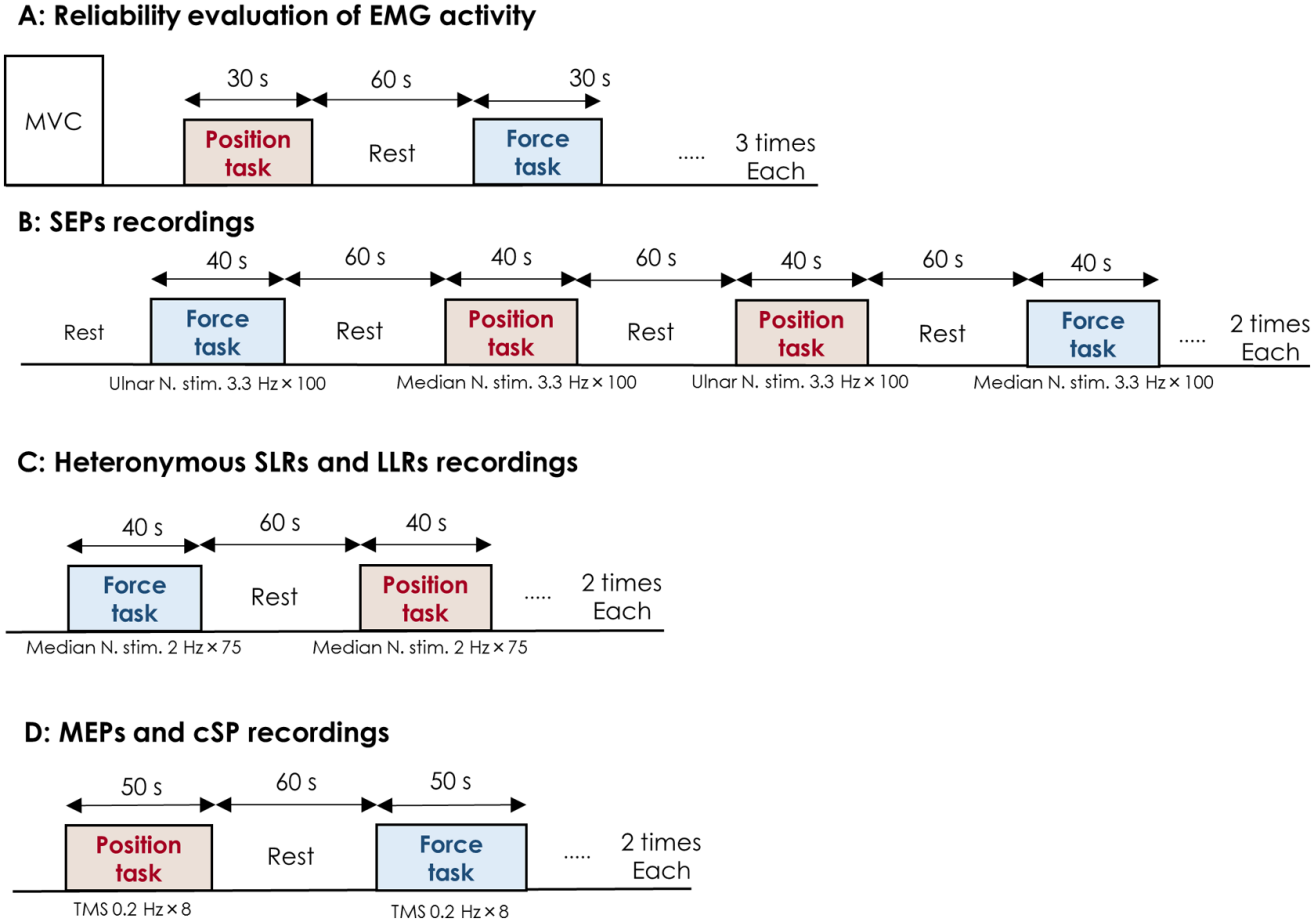
Experimental procedure. Experimental procedure for reliability evaluation of EMG activity (A), SEP recordings (B), SLR and LLR recordings (C) and MEP and cSP recordings (D). Median or ulnar nerve stimuli were delivered a total of 200 times during each task for SEP recordings. Median nerve stimuli were delivered a total of 150 times during each task for heteronymous SLR and LLR recordings. TMS was delivered 16 times during each task for MEP and cSP recordings.

The experimental session began with the performance of a MVC of the right first dorsal interosseous (FDI) muscle as the subject exerted an abduction force with the index finger. The MVC involved an increase in force from zero to maximum over 3 s and then holding that force for 3 s. At least three trials were performed, with subjects resting for 90 s between trials to minimize fatigue. We gave both visual and verbal feedback to subjects during MVC. If the MVC forces were within 5% of each other, the highest value was taken as the maximum, and used as a reference for submaximal contractions. If required, additional trials were performed until the 5% criterion was achieved. In addition, a single MVC of the abductor pollicis brevis (APB) was performed for electromyography (EMG) normalization. Then, for the FDI muscle, the subject performed a static contraction at 20% MVC force for the force task and at 10° abduction for the position task. Subjects executed the static contraction for approximately 90 s (divided into 3 blocks of 30 s) and were given a 60-s rest between each contraction to avoid fatigue (Fig. 2). The order of the two tasks was alternated across subjects. These data were used to evaluate the reliability of the EMG measurements across the two tasks.

Surface EMGs from the FDI and APB muscles were recorded using disposable silver-silver chloride surface electrodes. The recording and reference electrodes were placed over the muscle and tendon, respectively. EMG signals were amplified (×100) and bandpass filtered (5–500 Hz) with an amplifier (DL-140, 4 assist, Japan), and digitized at 10 KHz (PowerLab, AD Instruments). The data were recorded and stored for off-line analysis (Chart 7.3, AD Instruments) on a personal computer.

The following signals were recorded during the resting condition (as control) and the force and position tasks: MEPs from the right FDI in response to TMS over the left M1 area, SEPs in response to right ulnar or median nerve stimulation from the left C3′ (2 cm posterior to C3 of the International 10–20 system), and heteronymous reflexes (SLRs and LLRs) from the right FDI in response to right median nerve stimulation. MEPs in response to TMS, SEPs in response to ulnar nerve stimulation and heteronymous reflexes in response to median nerve stimulation were evaluated in separate trials. At each trial, subjects performed 2 blocks for each of the two tasks, with each block lasting approximately 40–50 s and separated by 60 s of rest to avoid fatigue (Fig. 2). The trial order was randomized across subjects. To minimize the influence of transient fluctuations in task mechanics on the reflex responses, stimuli were automatically delivered when force and position signals reached their respective targets and were maintained at a steady state for more than 1 sec.

### Recordings of SEPs

The ulnar (dominant nerve of FDI muscles) or median nerve (as control) was electrically stimulated at the wrist. Gold electrodes (10 mm in diameter) were placed at an inter-electrode distance of 10 mm, between the tendons of the palmaris longus and flexor carpi radialis muscles for median nerve stimulation, or beside the flexor carpi ulnaris muscle for ulnar nerve stimulation. The ground electrode was fastened to the skin between the stimulating and recording electrodes. Stimulus intensity was fixed at just above the motor threshold with a repetition rate of 3.3 Hz (Viking Quest, Nicolet, San Carlos, California, USA). The stimulus duration was 0.3 ms. The potentials were amplified and bandpass filtered (1–3,000 Hz), and 200 responses were averaged. SEP waveforms were evaluated for 100 ms, evaluations being performed at 50 ms before and 150 ms after stimulation. The amplification and recording unit also delivered the electrical stimuli. SEPs were recorded using silver-silver chloride electrodes (1.0 cm diameter). The recording electrode for SEPs was placed 2 cm posterior to C3, based on the International 10–20 system. A reference electrode was placed on the right earlobe.

### Recordings of Heteronymous reflexes (SLRs and LLRs)

Heteronymous reflexes in the FDI muscle were assessed by delivering an electrical stimulus to the median nerve. Stimulating and ground electrodes were placed as described above. Stimulus intensity was fixed at just below the motor threshold of the thenar muscles, with a repetition rate of 2 Hz and stimulus duration of 1 ms [43]. Heteronymous reflexes were recorded from the surface electrodes placed in belly-tendon fashion over the FDI muscle. The potentials were amplified and bandpass filtered (1–3,000 Hz), and 150 responses were averaged. The electrical stimulation and EMG recordings were performed by the same system, as described above.

The FDI muscle is innervated by the ulnar nerve; therefore, median nerve stimulation permitted the assessment of agonist responses to feedback transmitted by low-threshold afferents without concurrent activation of the antagonist muscle. This approach also avoided contamination of the H-reflex by F waves evoked by antidromic activation of homonymous motor axons [16], [44].

### MEP recordings

MEPs elicited by TMS were recorded from the right FDI muscle. TMS was performed using a standard double (‘figure-eight’) 70-mm coil connected to a monophasic Magstim 200 stimulator (Magstim, Carmarthenshire, UK). The coil was placed tangentially to the scalp with the handle pointing in a posterolateral direction that was 45° from the midline. We determined the optimal position for activation of the right FDI muscle by moving the coil over the presumed motor area of the hand in the M1 (approximately 4–6 cm lateral and 2 cm anterior to the vertex). The site where TMS of slightly suprathreshold intensity consistently elicited the largest MEPs in the FDI was marked as the motor hotspot. TMS thresholds were defined according to international guidelines [45], [46]. The active motor threshold (AMT) was defined as the lowest stimulus intensity required to produce MEPs of at least 200 µV amplitude in at least five of 10 consecutive trials, while the FDI muscle was activated at 5% of the MVC force. The stimulus intensity was adjusted by 1% of the maximum stimulator output (MSO) to determine these thresholds. TMS was delivered 16 times at 0.2 Hz, while each subject performed the force and position tasks with the index finger. The TMS intensity was set at >150% AMT (65–75% MSO), which has been shown to be optimal for obtaining a sufficient cSP duration [34], [39], [47].

The data were recorded and stored for off-line analysis (Scope, AD Instruments) on a personal computer.

### Data and statistical analysis

The average amplitude of the rectified EMG signal (aEMG) was calculated for a 0.5-s interval centered about the peak EMG of MVC trials for the FDI and APB muscles. Background activation of the intrinsic hand muscles were recorded during approximately 90 s of isometric contraction (20% MVC force and 10° abduction) for both the force and position tasks. The aEMG obtained over the entire time period of muscle contraction was normalized according to the value of the EMG amplitude at MVC (%EMG). Since there were two different load types, a single measure of the interclass correlation coefficient, ICC (2, 1), was used to measure the reproducibility of inter-load type EMG activity for the FDI and APB muscles.

Peak-to-peak amplitudes of SLRs, LLRs and MEPs were measured for analysis after excessive artifacts were automatically excluded by software. The duration of the cSP following TMS was measured in the contracting muscles as the interval from the stimulus artifact to the time of return of a continuous EMG amplitude that was more than 3-fold greater than the standard deviation of the background EMG noise at rest, and the average values were calculated for all cSP durations. The peak-to-peak amplitudes of the four cortical SEP components (N20, P25, N33 and P45) were also analyzed. The amplitude of each component was measured from the preceding peaks.

All data were expressed as the mean ± SEM. The significance of differences in EMG activity, the amplitudes of SLRs, LLRs and MEPs, and the duration of the cSP between the force and position tasks were tested using the Student’s paired-sample *t*-test. The amplitudes of the four cortical SEP components were statistically analyzed using repeated-measures analysis of variance (ANOVA) with the parameters of rest, force task and position task. The sphericity of the data was tested with Mauchly’s test, with Greenhouse–Geisser-corrected significance values being used when sphericity was lacking. Post-hoc analysis was performed with Bonferroni’s correction for multiple comparisons. The significance of differences was accepted at p<0.05 for all analysis.

## Results

### Inter-load type reproducibility of EMG activity

**Figure 3:**
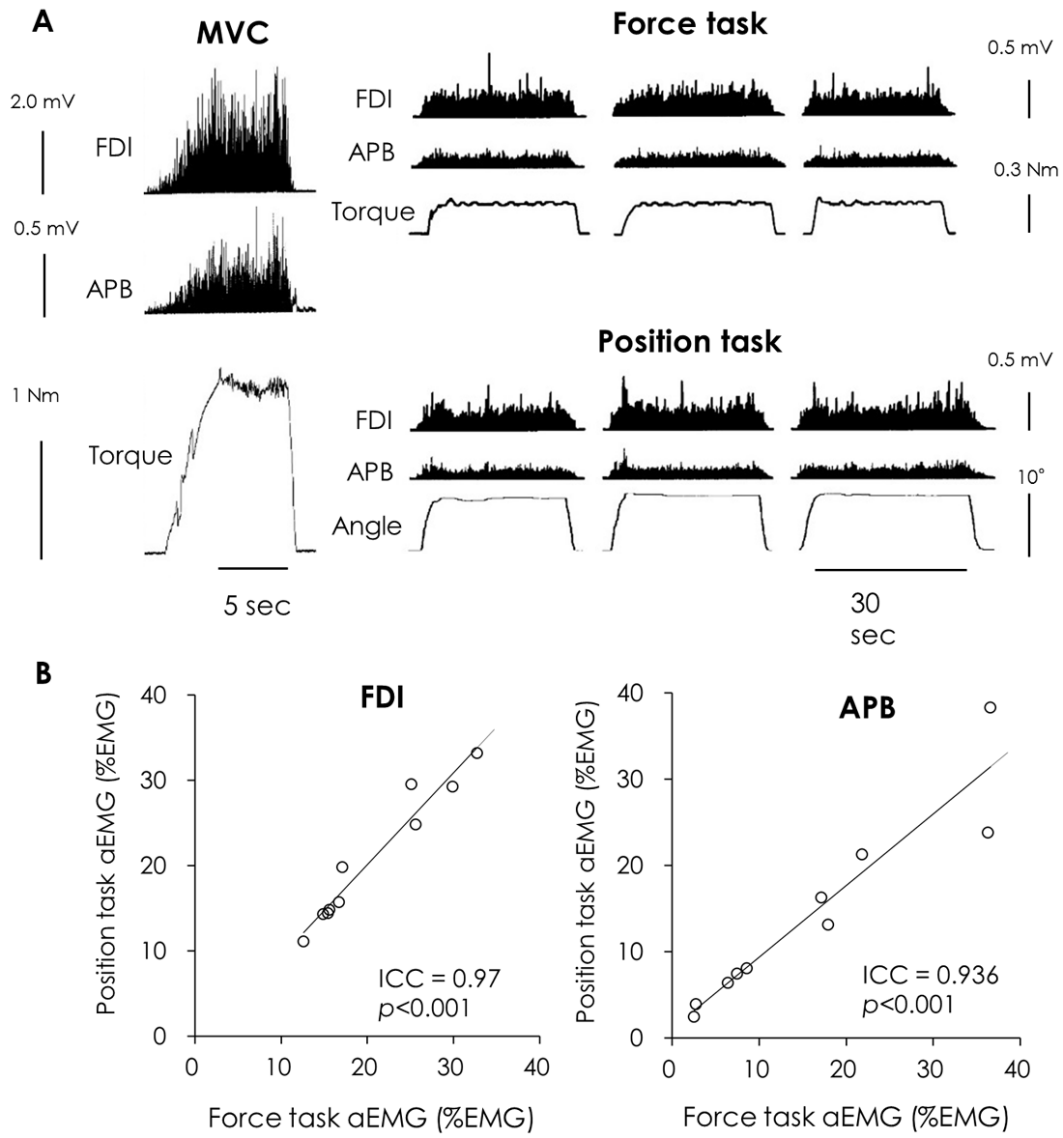
The EMG from the FDI and APB muscles, and the torque and/or angle waveforms recorded from a representative subject during MVC and isometric contraction when the subject maintained a constant finger force or position (A), and averaged amplitude of the rectified EMG signals (aEMG) from the FDI and APB muscles of the individual subjects for the two tasks (B). Regression analysis showing the relationship between the aEMG of the force and position tasks. Most of the data fell on the regression line, indicating a very high reproducibility between the force and position tasks.

Fig. 3 (A) shows the EMG, muscle torque and angle of metacarpophalangeal joint waveforms recorded from a representative subject during MVC and submaximal static contraction for the force and position tasks. The mean MVC and target force for all subjects was 2.0±0.4 Nm and 0.4±0.05 Nm, respectively. The aEMG of the FDI muscle during the two tasks performed for 30 s were similar between the force and position tasks (20.5±2.2% EMG and 20.7±2.4% EMG, respectively; *p* = 0.986). The aEMGs of the APB muscle were also similar during the force and position tasks (15.7±4.0% EMG and 14.1±3.5% EMG, respectively). Inter-load type reproducibility of EMG activity from the FDI [ICC (2, 1)  = 0.970] and APB [ICC (2, 1)  = 0.936] muscles were both excellent (Fig. 3B).

### Amplitude attenuation of SEPs during static contraction

**Figure 4:**
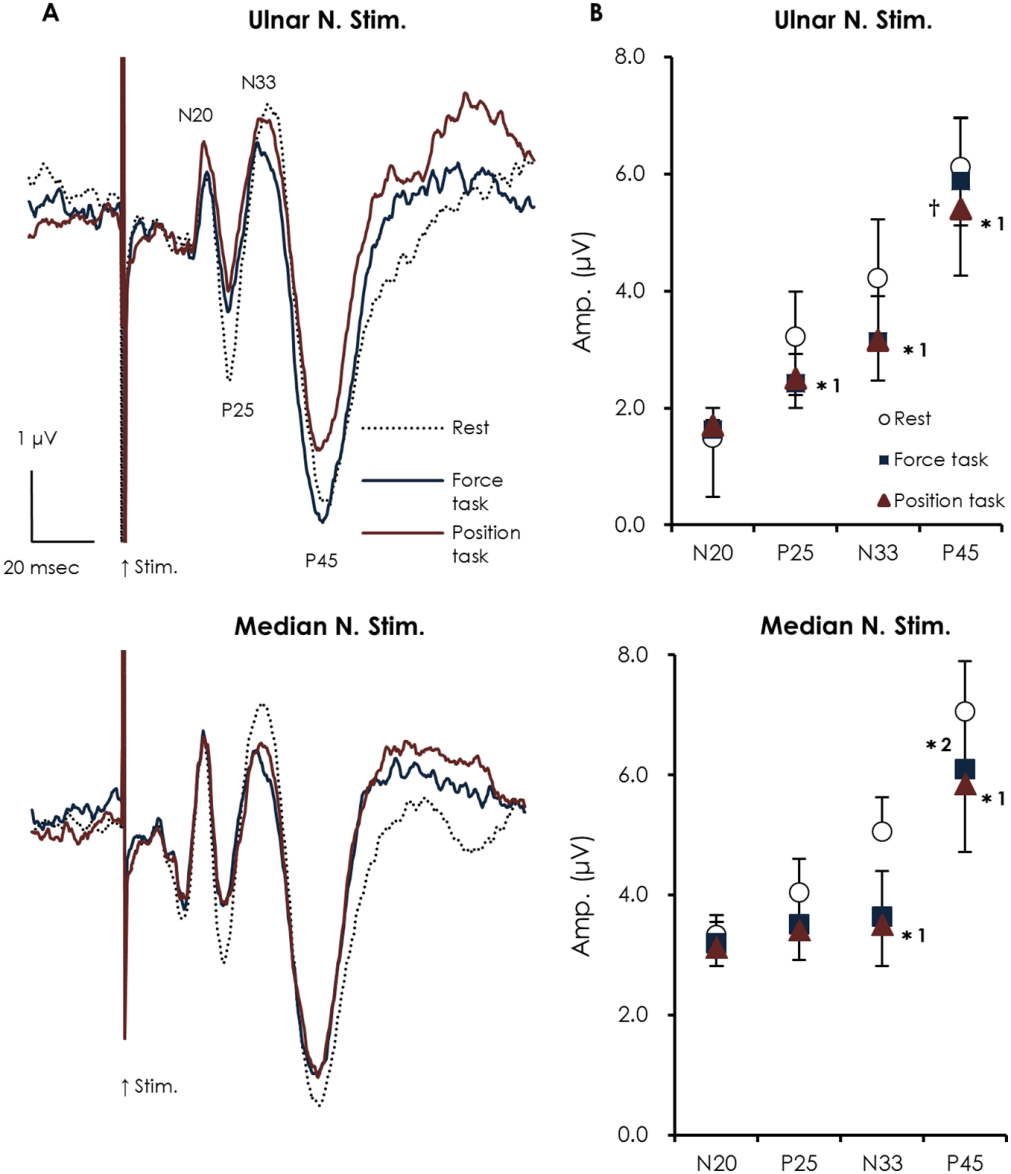
Averaged SEP waveforms recorded from C3′ (A) and the mean amplitude of each of the SEP components (B) at rest (dotted line) and during the force (blue line) and position tasks (red line) when either the right ulnar (upper) or median (lower) nerve was stimulated at the wrist. During ulnar nerve stimulation, the position task resulted in greater attenuation of the P45 amplitude than during the force task and rest conditions. However, when the median nerve was stimulated, load type did not significantly affect SEP gating (mean ± SEM). (position task vs. rest *^1^ *p*<0.05; force task vs. rest *^2^ *p*<0.05; position task vs. force task ^†^ *p*<0.05).

Fig. 4 (A) shows averaged waveforms of the SEPs recorded from the left parietal area by stimulating the right ulnar or median nerve during the resting condition (control) and during index finger abduction for both the force and position tasks.

The SEP amplitudes of N20 did not change significantly with static contraction during both ulnar nerve (F_2, 18_ = 1.464, p = 0.258) and median nerve stimulation (F_2, 18_ = 0.526, p = 0.6). One-way ANOVA on the data obtained during ulnar nerve stimulation revealed a significant effect of static contraction (*F*_1.18, 10.63_ = 6.531, *p* = 0.024) on the P25 component. Post-hoc analysis showed a significant difference between rest and the position task (3.2±0.6 µV and 2.5±0.5 µV, respectively; *p* = 0.046), while there were no significant differences between rest and the force task (*p* = 0.103), and between load types (*p* = 1.0). In contrast, no significant effect of voluntary contraction on P25 amplitude was observed during median nerve stimulation (F_2, 18_ = 2.664, *p* = 0.097). For the N33 component, one-way ANOVA on data obtained during ulnar nerve stimulation revealed a significant effect of static contraction (*F*_2, 18_ = 7.999, *p* = 0.03). Post-hoc analysis showed a significant difference between rest and the position task (4.2±0.9 µV and 3.2±0.7 µV, respectively; *p* = 0.017), whereas no significant difference existed between rest and the force task (*p* = 0.081), and between load types (*p* = 1.0). Similarly, using data obtained during median nerve stimulation, one-way ANOVA revealed a significant effect of static contraction (F_2, 18_ = 6.674, p = 0.007). Post-hoc analysis showed a significant difference between rest and the position task (5.1±1.0 µV and 3.5±0.7 µV, respectively; *p* = 0.045), while there was no significant difference between rest and the force task (*p* = 0.097), and between load types (*p* = 1.0). For the amplitude of the P45 component, one-way ANOVA revealed a significant effect of static contraction during both ulnar nerve (*F*_1.87, 12.87_ = 4.856, *p* = 0.036) and median nerve stimulation (*F*_1.68, 15.09_ = 25.236, *p*<0.001). When the ulnar nerve was stimulated, post-hoc analysis showed that the P45 amplitude during the position task was significantly smaller than both, those during the force task (5.4±0.7 µV and 6.0±0.8 µV respectively; *p* = 0.027) and at rest (5.4±0.7 µV and 6.2±0.8 µV respectively; *p* = 0.044), whereas there was no significant difference between rest and the force task (*p* = 0.311). Although the difference in the amplitude of P45 between the two tasks was very small, 9 of 10 subjects showed a tendency towards greater attenuation of P45 amplitude with the position task than the force task. Conversely, when the median nerve was stimulated, the P45 amplitude at rest was significantly larger than during the force (7.1±0.8 µV and 6.1±0.8 µV respectively; *p* = 0.003) and position tasks (7.1±0.8 µV and 5.8±0.7 µV respectively; *p*<0.001), although no significant effect of load type was observed (*p* = 0.737) (Fig. 4B).

### Amplitude of heteronymous SLRs and LLRs

**Figure 5:**
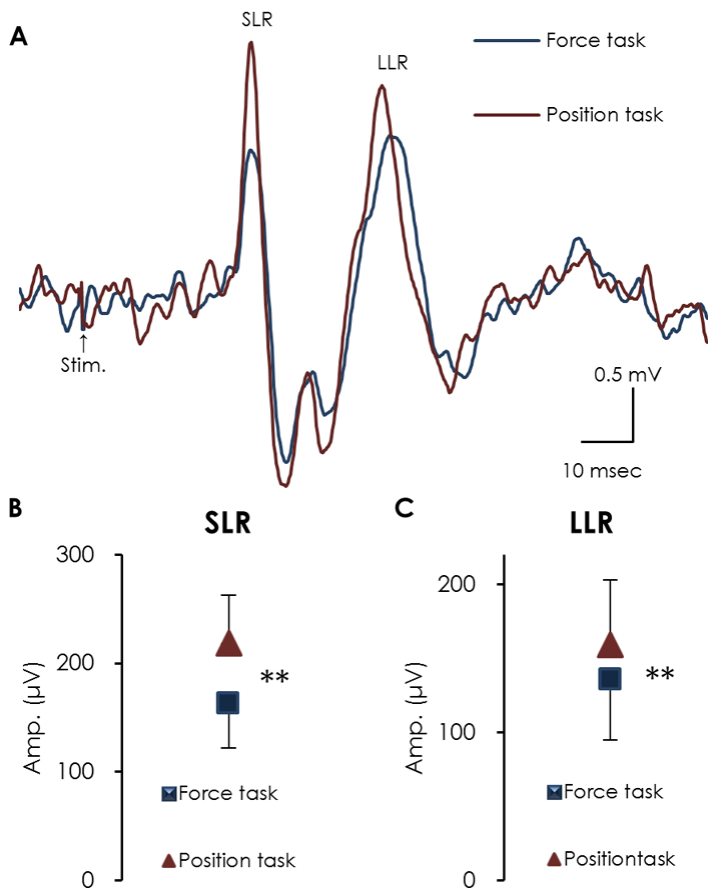
Averaged heteronymous SLR and LLR waveforms recorded from the FDI muscle by stimulating the median nerve during the force (blue line) and position tasks (red line) (A), and the mean amplitude of the SLRs (B) and LLRs (C). The amplitude of both the SLRs and LLRs during the position task was significantly larger than during the force task (mean ± SEM). (** *p*<0.01).

Fig. 5 (A) shows averaged waveforms of heteronymous SLRs and LLRs recorded from the FDI muscle in response to stimulation of the right median nerve during index finger abduction in the force and position tasks. The amplitude of heteronymous SLRs during the position task was significantly larger than that during the force task (218.8±44.1 µV and 162.6±40.8 µV, respectively; *p*<0.001) (Fig. 5B). Likewise, the amplitude of heteronymous LLRs during the position task was significantly larger than that during the force task (149.0±21.0 µV and 135.6±23.6 µV, respectively; *p* = 0.018) (Fig. 5C).

### Amplitude of MEPs and the cSP duration

**Figure 6:**
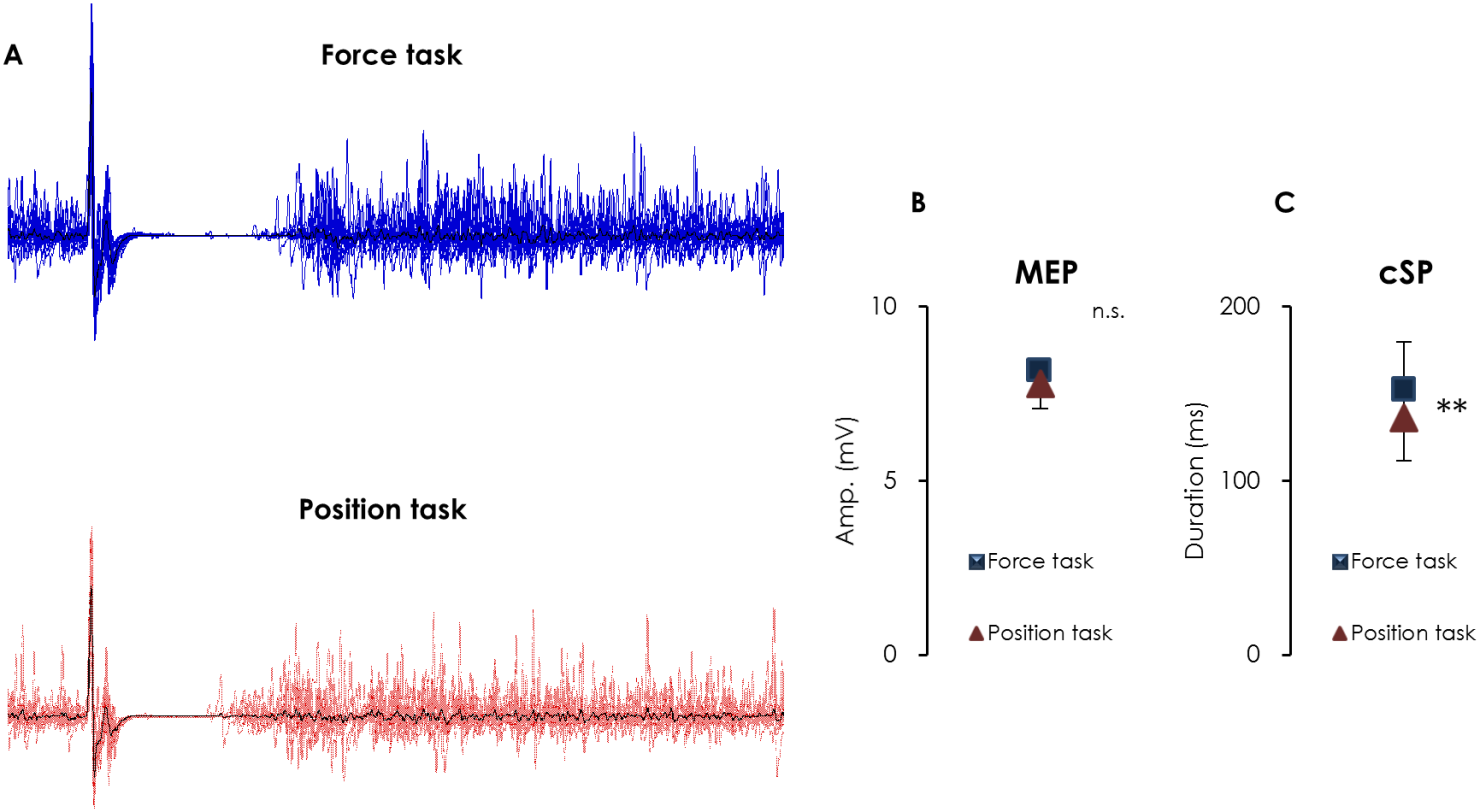
MEP waveforms and cSP (overlay of 16 trials) with TMS over the M1 recorded from a representative subject during the force (blue line) and position tasks (red line) (A), the mean amplitude of MEPs (B) and the duration of cSP (C) during the force and position tasks. The black lines in panel (A) show the average waveform of 16 trials. The force task resulted in a significantly longer cSP duration than the position task, while the amplitude of MEPs did not differ between the two tasks (mean ± SEM). (** *p*<0.01).

Fig. 6 (A) shows a representative overlay of the MEP waveforms and the cSP in response to TMS (delivered 16 times) recorded from the FDI muscle during the force and position tasks. The amplitude of MEPs did not differ between the force and position tasks (8.2±2.1 mV and 7.8±2.3 mV, respectively; *p* = 0.255) (Fig. 6B); however, the position task resulted in a significantly shorter cSP duration compared with the force task (135.8±30.1 ms and 152.1±32.6 ms, respectively; *p* = 0.013) (Fig. 6C).

## Discussion

A novel observation of our study was that, during FDI muscle contraction, the reduction of SEP amplitude (P45) was significantly larger during the position task than that during the force task or at rest when the ulnar nerve (which innervates the FDI muscle) was stimulated, while no significant effect of load type was observed when the median nerve was stimulated. In concurrence with previous reports [16], [18], the amplitude of the heteronymous SLR was significantly greater for the position task than for the force task. In addition, the duration of the cSP during the position task was significantly shorter than during the force task, while the amplitude of MEPs did not differ between tasks. The larger amplitude of the heteronymous SLR and larger gating effect of SEP amplitude (P45) during the position task suggests that maintaining the position of the index finger while supporting a constant load requires more proprioceptive information, which enhances gating of P45. The shorter duration of the cSP during the position task may be attributable to the larger amplitude of heteronymous SLRs.

The SEP gating phenomenon is not only due to competition between the input from the presented stimulus and the afferent proprioceptive feedback caused by movement itself (centripetal gating), but also involves the centrifugal influence of motor centers on the synapses of the sensory pathways (centrifugal gating), as suggested by the reduction of SEPs when the stimulus precedes the onset of active movement [24] and pre-movement [48]–[50]. Due to the fact that the gating phenomenon may occur anywhere along the ascending sensory pathway or in the cerebral cortex, we need to address the mechanisms causing the differences in attenuation of the P45 component of SEPs between position and force tasks, considering both centripetal and centrifugal gating mechanisms. There are few studies on SEP gating in which each subject generated the same muscle torque by visual or auditory feedback during isometric finger contraction. Touge et al. [27] examined the effects of aging on SEP modification by static muscle contraction, and demonstrated larger attenuation of the N33 and P45 SEP components in aged subjects compared with younger subjects. Older subjects are reported to require activation of more brain areas, such as the pre-motor areas, cerebellum and pre-supplementary motor areas, for voluntary movements [51]. These age-related changes could, reportedly, contribute to the differential gating effects. Our study, however, demonstrated no effects of aging on the physiological mechanisms of SEPs gating. Yet, it is entirely fair to say that these two studies have something in common in terms of the need for greater proprioceptive information to induce attenuation of middle SEP components (N33 and P45) during static muscle contraction, as evinced by the fact that attenuation of the P45 component of SEP when the median nerve was stimulated was comparable between the two tasks. This could be interpreted to indicate that the gating effect is attributable to involuntary APB muscle contraction that occurs to stabilize index finger abduction, working as a functional agonist [52], [53]. The subjects were very careful about the contraction levels of the FDI muscles, which might require more proprioceptive information to maintain the position of the index finger while supporting a constant load. Therefore, we inferred that greater proprioceptive input and/or more cortical activation is produced during the position task, and this explains the greater gating (P45) compared to the force task only when the ulnar nerve, but not the median nerve, was stimulated. Another possible explanation for the attenuation of P45 could be the effects of activity in the posterior parietal cortex (PPC) [54]–[56]. Furthermore, Inui et al. [54] proposed a hierarchical scheme of somatosensory processing from area 3b (peaking at 21–30 ms) to area 1 (peaking at 25–34 ms) and the PPC (peaking at 29–37 ms). This suggests the possibility that the difference in sensorimotor modulation between the two tasks is due to the highly hierarchical scheme of somatosensory processing, which induces larger gating of the P45 component of SEPs in the position task.

In agreement with previous reports [16], [18], our study showed that the amplitude of the heteronymous SLR was significantly greater for the position task than for the force task. To avoid concurrent activation of FDI and its antagonist, and to limit contamination of the recording by F-waves [44], many investigators use a heteronymous pathway to induce an H-reflex in the FDI [16], [18], [43]. Since the F-wave represents the response of motor neurons to an antidromic volley [57], [58], it does not occur when activating a heteronymous pathway. Similarly, in our study, the larger heteronymous SLR amplitude during the position task suggests that the heteronymous monosynaptic Ia facilitation during the position task was greater than that during the force task.

In the present study, the amplitude of MEPs did not differ between the force and position tasks. This is in agreement with previous studies, in which the target muscles were the FDI [33] and biceps brachii muscles [10]. Based on the principle of recruitment order, when EMG activity increases, larger pyramidal neurons in the M1 or α motor neurons are recruited, which result in larger MEPs [31]. Our results showed high reproducibility of inter-load type EMG activity (force and position tasks) during isometric contraction of the FDI muscle. Therefore, our result that the amplitude of MEPs did not differ between the two tasks is to be expected. While high stimulus intensity is optimal for obtaining a long cSP, it also produces very large MEPs and likely stimulates the cortex and deeper white matter structures (by direct activation of corticospinal axons and D-waves) [59], [60]. Thus, large MEPs might not be solely representative of cortical excitability within the M1, and, consequently, they might not be sensitive to subtle differences in cortical excitability between the tasks in the present study.

The cSP refers to an interruption of voluntary muscle contraction by TMS of the contralateral M1. It is generally thought that although a spinal inhibitory mechanism may contribute to the early part of the cSP up to its first 50 ms, its later part is generated exclusively by inhibition that originates within M1 [61]. Therefore, the cSP can be considered as a probe of motor cortical inhibition. In this study, the cSP duration was shorter with the position task than with the force task, whereas the amplitude of MEPs did not differ between the two tasks. The concept that the MEP and cSP are generated by different physiological mechanisms [38] is supported by some previous studies. The cSP threshold is usually slightly lower than the MEP threshold [62]. The cSP duration is largely a linear function of TMS intensity [37], [39], [63], with the stimulus intensity, but not the level of contraction, affecting the cSP duration [34], [47]. These results may partly explain our finding that the cSP during the position task was shorter than that during the force task, while the amplitude of MEPs did not differ between the two tasks. Further, our results suggest that inhibitory input from M1 to the alpha motor neuron pool is smaller in position tasks than in force tasks. Meanwhile, our results appear to differ from those of a previous study [10] in which the duration of the cSP was comparable between force and position tasks in an elbow flexion task. Besides differences in agonist muscles properties (upper-limb versus intrinsic muscle of the hand), one possible explanation for this discrepancy is the use of nonfocal and lower current density round stimulation coil [64] [65] in the early study, which may have resulted in additional facilitatory/inhibitory interaction with the descending volley in cortico-spinal pathway. However, the reason for this discrepancy is still debatable.

Conditioning electrical stimulation of cutaneous afferents shortens the cSP. This effect shows a topographic gradient and is most pronounced if a cutaneous digital nerve adjacent to the target muscle is stimulated [66]. This suggests that the cSP is involved in sensorimotor modulation that is somatotopically organized. Sowman et al. [67] reported that the time course of stretch reflex suppression approximately matched the duration of the masseter cSP induced by TMS during voluntary activation. In addition, Binder et al. [40] reported that in vibrating muscles (i.e., enhanced Ia afferent activity), the cSP duration tended to become shorter, whereas the amplitude of MEPs remained unchanged. We must avoid confusing corticobulbar control of presynaptic inhibition of Ia afferent terminals in the trigeminal motor system with that of the motor neuron pool of limbs and vibrating muscles that supports a compliant load. However, there is the possibility that sensorimotor integration results in the shorter duration of the cSP during the position task, which is associated with the larger amplitude of heteronymous SLRs, and can be used as a surrogate marker for presynaptic Ia inhibition [68].

In conclusion, the most relevant findings of our study are as follows: reduction of the P45 amplitude was significantly larger during the position task than the force task when the ulnar nerve, but not the median nerve, was stimulated, and the duration of the cSP during the position task was significantly shorter than that during the force task. These findings suggest that sensorimotor modulations differ with the load type during constant finger force or position.
